# Trends and profiles of acute poisoning cases: a retrospective analysis

**DOI:** 10.3389/fpubh.2023.1235304

**Published:** 2023-09-05

**Authors:** Shifang Liu, Lijing Ling, Jin Ma, Hua Yuan, Zhiqiang Guo, Qiupeng Feng, Xiaohua Xia

**Affiliations:** Department of Emergency Medicine, Affiliated Kunshan Hospital of Jiangsu University, Kunshan, Jiangsu, China

**Keywords:** acute poisoning, suicide, retrospective study, sociodemographic profiles, temporal analysis

## Abstract

Acute poisoning is a significant public health concern. This retrospective study investigates trends in acute poisoning cases and explores the clinical and sociodemographic profiles associated with this condition. Medical data from 859 hospitalized patients diagnosed with acute poisoning between January 2017 and December 2022 were comprehensively analyzed. The descriptive statistical analysis revealed that 360 patients had underlying diseases, with depression being the most prevalent among them. Furthermore, urban areas accounted for 87.2% of the acute poisoning cases, indicating a higher incidence compared to rural areas. The substances implicated in acute poisoning incidents varied, with drugs of abuse being the most common (53.2%), followed by pesticides (22.2%), carbon monoxide (11.8%), and alcohol (5.4%). Suicide attempt/suicide emerged as the leading cause of acute poisoning incidents, accounting for 75.9% of cases, while poisoning accidents predominantly occurred within the home setting. Through chi-square tests, it was determined that risk factors for suicide attempt/suicide included female gender and underlying medical conditions. Temporal analysis showed that the total number of acute poisoning cases increased from 2017 to 2019 and decreased from 2019 to 2022. Notably, suicide-related cases exhibited an upward trend, with suicide attempt/suicide accounting for over 80% of all acute poisoning cases after 2020. This study contributes valuable insights into the trends, profiles, and risk factors associated with acute poisoning cases.

## Introduction

Acute poisoning is a condition characterized by sudden and severe exposure to a toxic substance (1–3). The symptoms of acute poisoning vary depending on the type and dosage of the toxic substance and may include nausea, vomiting, headache, coma, and even death (3, 4). This condition is a frequent cause of emergency department visits and hospitalizations, accounting for up to 10% of the emergency department’s caseload (5). According to a study conducted by the Pesticide Action Network North America (PANNA), approximately 385 million cases of acute poisoning occur worldwide each year. Among adults, the leading causes of acute poisoning are drugs, followed by carbon monoxide and alcohol (5, 6). Moreover, there are numerous substances, including organophosphates, that have the potential to induce acute poisoning (3, 7–10).

Most admissions of acute poisonings to the emergency department are attributed to deliberate self-harm rather than accidental incidents. Between 2015 and 2019, there was a notable 26% increase in the number of individuals attempting suicide through the ingestion of toxic substances or medication overdose (11). Additionally, a separate study indicates a significant surge in the rates of attempted suicides and suicides involving poisoning among young people during the COVID-19 pandemic (12). These emerging patterns underscore the imperative for further research aimed at comprehending the underlying risk factors associated with the choice of poisoning as a suicide method. Furthermore, there is a pressing need to develop effective prevention and intervention strategies to mitigate the risk of suicide related to poisoning.

It is worth mentioning that the etiology of acute poisoning has experienced notable transformations in recent years (13, 14). Previously prevalent causes of poisoning, such as paraquat and rodenticides, have become increasingly rare in emergency department settings (14, 15). Therefore, it is imperative to investigate the shifts in the causes of acute poisoning in recent years to enhance the efficient allocation of emergency medical resources.

In this study, we conducted a retrospective analysis encompassing patients diagnosed with acute poisoning in the emergency department between January 2017 and December 2022. The objective of this investigation was to provide a comprehensive depiction of the clinical and sociodemographic profiles associated with acute poisoning.

## Materials and methods

### Study design, setting, and participant

We performed a retrospective cross-sectional study on cases of acute poisoning treated in the emergency department of the Affiliated Kunshan Hospital of Jiangsu University, which is the sole Grade III general hospital in Kunshan. Annually, the emergency department handles over 150,000 patients, with more than 6,500 requiring rescues. To obtain data, we extracted medical information from the hospital’s information management system, focusing on 859 hospitalized patients diagnosed with acute poisoning between January 2017 and December 2022. The diagnosis of acute poisoning was based on the patient’s history, physical examination, and routine and toxicological laboratory evaluations. We excluded cases that lacked clinical information, involved non-acute poisoning, or pertained to individuals under the age of 14 (typically referred to the Children’s Hospital). The collected medical information encompassed sociodemographic factors (age, gender, underlying diseases, and residential area), clinical aspects (type of poisonous substances, place of poisoning, and cause of poisoning) and outcome details. Poisonous substances were classified as drugs, pesticides, carbon monoxide (CO), alcohol or other substances. The causes of poisoning were categorized as either suicide attempt or accident, while the place of residence was classified as rural or urban.

### Statistical analysis

IBM SPSS version 26 software was used for statistical analysis. Descriptive statistical method was employed to evaluate the data. The difference between suicide and accident cases was assessed using the chi-square test. Furthermore, comparisons were made to analyze trends in population distribution, poisonous substances, and causes of acute poisoning over a span of 6 years. A significance level of *p* < 0.05 was utilized to indicate statistically significant differences.

## Results

### Basic characteristics of patients with acute poisoning

A total of 859 cases of acute poisoning (age ≥14 years old) were extracted from the electronic hospital information system of the Affiliated Kunshan Hospital of Jiangsu University in Kunshan, China, spanning the period from January 1, 2017, to December 31, 2022. The key characteristics of the enrolled patients with acute poisoning are summarized in Table 1. Of these cases, 357 were males and 502 were females, resulting in a gender ratio of 0.71:1. The proportion of acute poisoning patients between 18 and 45 years old was the highest (57.4%). Out of the total number, 360 cases presented with a history of underlying diseases, such as depression, cancer, cardiovascular disease, nervous system disease, diabetes, comorbidity and other ailments. Among those with underlying conditions, depression was the most prevalent, accounting for 279 cases. The incidence of acute poisoning was significantly higher in urban areas compared to rural areas, with urban areas comprising 87.2% of the cases and rural areas accounting for 12.8%. Notably, the vast majority of patients (98.8%) survived after receiving treatment.

**Table 1:** Basic characteristics of hospitalized patients with acute poisoning.

	Number of cases	Percentage (%)
**Gender**		
Male	357	41.6
Female	502	58.4
**Age**		
14 < age < 18	51	5.9
18 ≤ age < 45	493	57.4
45 ≤ age < 65	204	23.7
65 ≤ age < 80	68	7.9
Age ≥ 80	43	5.0
**Underlying diseases**		
No	499	58.1
Depression	279	32.5
Cancer	17	2.0
Cardiovascular disease	16	1.9
Nervous system disease	9	1.0
Diabetes	4	0.05
Comorbidity	26	3.0
Other diseases	9	1.0
**Place of residence**		
Rural	110	12.8
Urban	749	87.2
**Outcome**		
Death	10	1.2
Survive	849	98.8
**Total**	859	100.0

### Poisonous substances, cause of poisoning and place of poisoning

Drugs of abuse were present in 457 patients (53.2%), pesticides in 191 (22.2%), CO in 101 (11.8%), and alcohol in 46 (5.4%) (Table 2). The majority of drug poisonings were linked to the consumption of antidepressants and sleeping pills. Notably, paraquat, a commonly reported toxic agent in previous years, accounted for a reduced number of acute poisoning cases in recent years. The cause of poisoning was categorized as either suicide or accident (Table 2), with suicide attempt accounting for the majority of acute poisoning incidents (75.9%). Poisoning accidents predominantly occurred within the confines of the patients’ homes (93.8%) (Table 2).

**Table 2:** Distribution of poisonous substances, poisoning causes, and poisoning locations.

	Number of cases	Percentage (%)
**Poisonous substance**		
*Pesticides*		
Total	191	22.2
Organophosphorus	89	10.4
Pyrethroids	52	6.1
Paraquat	2	0.02
Diquat	12	1.4
Rat poison	26	3.0
Others	12	1.4
*Drugs*		
Total	457	53.2
Sleeping pill	160	18.6
Antidepressant medications	125	14.6
Sleeping pill & antidepressant medications	100	11.6
Antipyretics	25	2.9
Others	47	5.5
*Alcohol*	46	5.4
*Co*	101	11.8
*Others*		
Total	64	7.5
Daily chemical products	36	4.2
Poisonous mushroom	15	1.7
Others	13	1.5
**Cause of poisoning**		
Suicide	652	75.9
Accident	207	24.1
**Place of poisoning**		
At home	806	93.8
Not at home (school, medical facility, outdoors etc.)	53	6.2

### Risk factors for suicide attempt

The acute poisoning patients were categorized into two groups based on the cause: suicide (75.9%) and accident (24.1%). Significant differences were observed between these groups in terms of gender (*χ*^2^ = 28.487, *p* < 0.0001), age (*χ*^2^ = 18.621, *p* = 0.001), and underlying diseases (*χ*^2^ = 158.045, *p* < 0.0001) (Table 3). Being female, adolescent and older adult, and having underlying medical conditions were risk factors for suicide attempts. However, no significant associations were found between place of residence and the incidence of acute poisoning.

**Table 3:** Risk factors of suicide using chi-square test.

Factor	Non-suicide	Suicide	*χ* ^2^	*p*-value
**Gender**				
Male	119	238	28.487	<0.0001
Female	88	414		
**Age**				
<18	6	45	18.621	0.001
18 ≤ age < 45	132	361		
45 ≤ age < 65	57	147		
65 ≤ age < 80	8	60		
Age ≥ 80	4	39		
**Underlying diseases**				
None	198	301	158.045	<0.0001
Yes	9	351		
**Place of residence**				
Rural	32	78	1.720	0.190
Urban	175	547		

### Pattern in poisoning characteristics from 2017 to 2022

**Figure 1:**
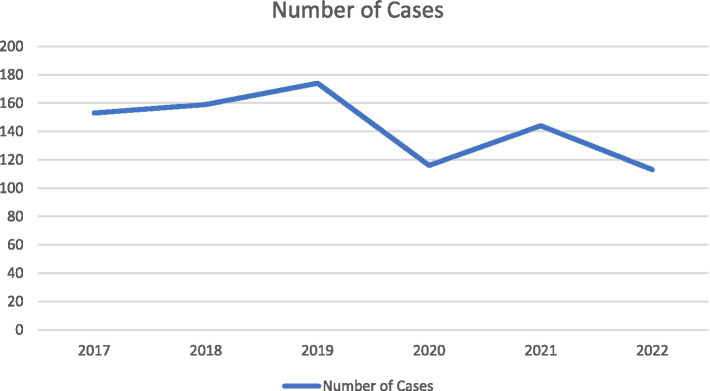
Time-varying curve of the number of acute poisoning cases.

**Figure 2:**
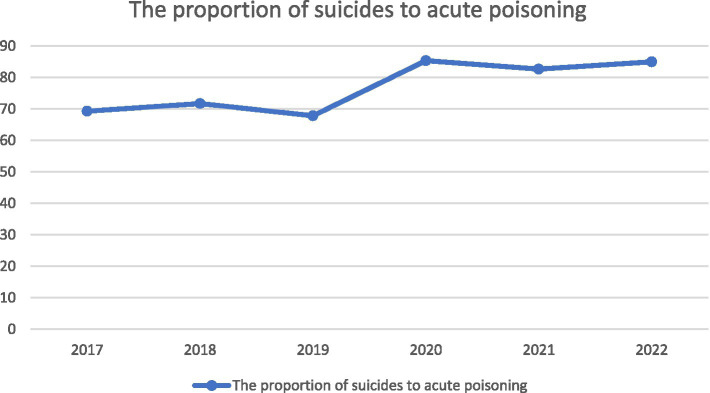
Time-varying curve of the proportion of suicides to acute poisoning cases.

**Figure 3:**
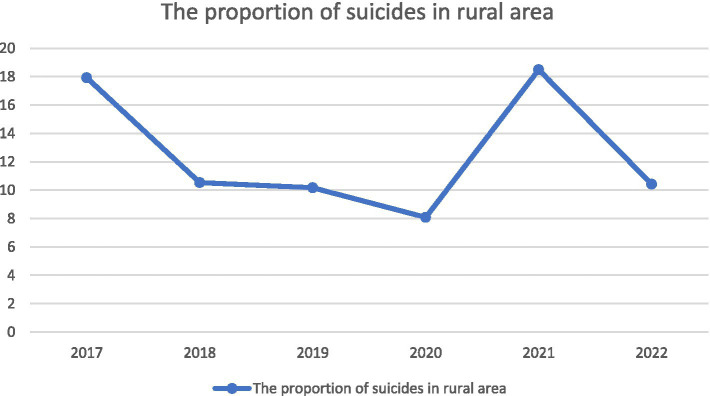
Time-varying curve of the proportion of suicide cases in rural areas.

From 2017 to 2022, there was an initial increase followed by a subsequent decrease in the number of acute poisoning cases (Figure 1). Among acute poisoning patients, there was an upward trend in the proportion of cases attributed to suicide, with suicide accounting for over 80% of cases after 2020 (Figure 2). The admission rates of rural patients to hospitals for suicide showed a fluctuating pattern, initially decreasing, then increasing, and finally decreasing again (Figure 3).

## Discussion

Acute poisoning is a major public health problem and an important reason for hospital emergency department visits (16–19). In this study, we investigated the clinical and sociodemographic profiles associated with acute poisoning cases, shedding light on the trends and risk factors in Chinese population. Among the 859 hospitalized patients diagnosed with acute poisoning, the majority of cases were female. The presence of underlying medical conditions, particularly depression, was identified as a significant risk factor for suicide attempt/suicide-related acute poisoning incidents. This finding highlights the association between mental health disorders and acute poisoning incidents. The substances involved in acute poisoning incidents varied, with drugs of abuse being the most common (53.2%), followed by pesticides (22.2%), carbon monoxide (11.8%), and alcohol (5.4%). It is worth noting that the incidence of poisoning due to paraquat, a previously common toxic agent, has decreased in recent years. This shift in poisoning patterns necessitates ongoing surveillance and adaptation of emergency medical resources to effectively respond to emerging toxicological challenges.

Suicide emerged as the leading cause of acute poisoning incidents, accounting for 75.9% of the cases. Furthermore, the temporal analysis revealed an upward trend in suicide-related cases, particularly after 2020. Notably, this timeframe aligns with the onset of the COVID-19 pandemic. The COVID-19 pandemic significantly altered daily routines and social interactions, amplifying the influence of the home environment on mental health. Lockdowns, quarantine measures, and remote work arrangements confined individuals to their homes, potentially exacerbating feelings of isolation and distress (20–23). This surge in psychological distress is closely linked to a substantially elevated risk of suicide and self-harm incidents amid the COVID-19 pandemic (20, 22, 24). The COVID-19 pandemic-induced uncertainties and anxieties further underscored the importance of home-based prevention strategies. Therefore, it is critical to develop effective prevention and intervention strategies to reduce the risk of suicide during the COVID-19 pandemic and during its subsequent years.

Gender differences in acute poisoning cases emerged as an essential factor to consider in suicide-related incidents. We found that females were more likely to be involved in acute poisoning incidents with suicidal intent compared to males. This aligns with previous research indicating gender-specific patterns in suicidal behavior (25–27). To assess the relationship between gender and suicide, Miranda-Mendizabal et al. (27) conducted a meta-analysis that included 67 studies, demonstrating a higher risk of suicide attempts in women compared to men (OR 1.96, 95% CI 1.54–2.50). Additionally, the study revealed that female-specific suicide risk factors, including depressive symptoms mentioned in our study, contributed to this higher risk. In the Chinese population, women are also identified as a significant risk factor for suicide. In a more recent study, Zheng et al. (26) prospectively included 237 patients treated for poisoning between May 2021 and May 2022, and their findings supported our results, with being female and anxious being significant risk factors for suicide. Thus, understanding these gender differences is essential for the development of gender-specific prevention and intervention strategies.

This study has some limitations. Firstly, this is a single center, retrospective study, which may limit the generalizability of the findings to other settings. Possibly shedding light on enhancing the representativeness of the results, future studies should consider a multi-center approach. Secondly, this study only looked at acute poisoning cases in the last 6 years. A longer time span of cases is needed to determine trends in the characteristics of acute poisoning.

## Conclusion

This study provides valuable insights into the trends, profiles, and risk factors associated with acute poisoning cases, with a particular emphasis on suicide-related incidents and gender differences. The findings emphasize the importance of addressing mental health issues, particularly depression, in individuals at risk for suicide-related acute poisoning. By revealing changing toxicological patterns, identifying vulnerable populations, and recognizing urban-rural disparities, the study lays the groundwork for evidence-based public health initiatives to address the multifaceted challenge of acute poisoning.

## Data availability statement

The raw data supporting the conclusions of this article will be made available by the authors, without undue reservation.

## Ethics statement

Ethical approval was not required for the studies involving humans because this was a retrospective study and did not include personal information about the cases. The studies were conducted in accordance with the local legislation and institutional requirements. Written informed consent for participation was not required from the participants or the participants’ legal guardians/next of kin in accordance with the national legislation and institutional requirements because this was a retrospective study and did not include personal information about the cases.

## Author contributions

SL, LL, JM, HY, ZG, and QF collected and analyzed the data. JM and XX designed this study. All authors contributed to the article and approved the submitted version.

## Conflict of interest

The authors declare that the research was conducted in the absence of any commercial or financial relationships that could be construed as a potential conflict of interest.

## Publisher’s note

All claims expressed in this article are solely those of the authors and do not necessarily represent those of their affiliated organizations, or those of the publisher, the editors and the reviewers. Any product that may be evaluated in this article, or claim that may be made by its manufacturer, is not guaranteed or endorsed by the publisher.
